# Disruption of the ubiquitin-mediated proteolysis pathway: a study of seed aging in *Saposhnikovia divaricata* caused by UBC1 gene family suppression

**DOI:** 10.1186/s12870-026-09258-3

**Published:** 2026-06-23

**Authors:** Chongyang Yang, Jiayuan Xing, Weixu Han, Jinping Gao, Haomin Zhang, Yuran Wang, Shuangli Liu

**Affiliations:** 1 College of Traditional Chinese Medicine, Jilin Agricultural University, Changchun, 130118 China; 2 Engineering Research Center of Ginseng, Changchun, 130118 China

**Keywords:** *Saposhnikovia divaricata*, Seed aging, Reactive oxygen species (ROS), E2 ubiquitin-conjugating enzyme (UBC1), E1 ubiquitin-activating enzyme (UBA1)

## Abstract

**Background:**

*Saposhnikovia divaricata* (Turcz.) Schischk. is a perennial herb whose seed aging during storage significantly reduces germination rates, limiting industrial-scale production. Reactive oxygen species (ROS)-induced oxidative damage is a key driver of seed aging, but the underlying mechanisms in *Saposhnikovia divaricata* remain unclear.

**Results:**

Suppression of the UBC1 gene family reduces the activity of ubiquitin-conjugating enzymes, leading to dysfunction of the ubiquitin-mediated proteolysis pathway, which in turn decreases protein degradation efficiency and causes the accumulation of damaged proteins. Transcriptome analysis revealed predominant downregulation of genes crucial for seed physiological maintenance. By the fourth year of storage, germination dropped sharply to 30.67%, accompanied by embryo cavitation. Downregulation of ribosome pathway genes hindered ribosome assembly and protein synthesis, while suppression of endoplasmic reticulum protein processing genes led to unfolded/misfolded protein accumulation and intensified cellular stress, accelerating aging. Proteomic analysis showed increased total differential and antioxidant-related proteins. ROS content fluctuated with storage time: peroxyl radicals peaked in year two (5.68 RFU/mg), whereas hydroxyl radicals and hydrogen peroxide were highest in year four (0.0655 pg/mL and 0.0946 pg/mL, respectively), with significant differences across periods. Elevated membrane-related proteins, increased electrical conductivity, and malondialdehyde content (maximum 54.30 nmol/g at year four) confirmed oxidative membrane damage. ROS-induced stress promotes protein misfolding, and reduced UBC1 expression is associated with impaired clearance of misfolded proteins by the ubiquitin-mediated proteolysis pathway.

**Conclusions:**

This study provides the first integrated transcriptomic and proteomic insight into UBC1 deficiency–mediated seed aging in *Saposhnikovia divaricata*. The findings enhance molecular understanding of seed aging and offer new directions for improving seed storage and viability.

**Supplementary Information:**

The online version contains supplementary material available at https://doi.org/10.1186/s12870-026-09258-3.

## Background

*Saposhnikovia divaricata* (Turcz.) Schischk., a perennial Apiaceae species, is a high-demand crude drug in *traditional Chinese medicine (TCM)*. Designated as a Category III nationally protected wild medicinal resource, it serves as a core component in TCM formulations targeting exterior syndromes, including Fangfeng Tongsheng Pills, Chuanxiong Chatiao Powder, and Yupingfeng Granules [1–4]. The escalating market demand has accelerated overexploitation of wild *Saposhnikovia divaricata* populations. Scaling up standardized cultivation protocols to achieve yield optimization now represents a critical agricultural imperative [5]. During cultivation, *Saposhnikovia divaricata* is primarily established by direct seeding. After sowing, germination generally takes more than one month, and some seeds may germinate in the following spring [6]. The germination rate of *Saposhnikovia divaricata* seeds stored for more than 12 months drops to 55% [7]; germination declines with seed aging. It is evident that seed quality substantially affects the sustainable development of the *Saposhnikovia divaricata* industry.

Germination rate is a fundamental indicator for evaluating seed quality variation and is collectively influenced by multiple external factors, such as storage temperature and storage duration [8–10]. A study on *Pinus densiflora* seeds stored for 20 years at different temperatures demonstrated that elevated storage temperatures exacerbate cellular membrane damage, resulting in increased exosmosis of cell sap and a decline in germination rate [11]. Conversely, low-temperature storage effectively preserves seed viability; for example, storing *Pisum sativum* seeds at 4 °C maintained optimal germination rate, hypocotyl length, and radicle length, thereby effectively conserving the physiological activity and structural integrity of the seeds [12]. During long-term storage, seeds of *Bambusa arundinacea* progressively accumulate reactive oxygen species (ROS). These ROS damage cellular structures and functions, thereby impairing seed germination and seedling development, leading to a continual decline in germination rate with increasing storage duration [13]. The internal physiological mechanisms underlying the decline in germination due to seed aging induced by external factors are typically associated with disrupted oxidative homeostasis [14]. Studies have demonstrated that ROS play a pivotal role in regulating seed dormancy and germination, and they are also the primary endogenous factor contributing to seed aging and damage. The magnitude of ROS levels directly dictates whether seeds transition into the germination stage or progress towards senescence [15, 16]. In model plants such as *Arabidopsis thaliana*, mitochondria are the principal source of ROS during the early imbibition phase. Upon radicle protrusion, ROS accumulate within the cell nucleus, accompanied by nuclear enlargement and chromatin decondensation [17]. Meanwhile, studies have also found that abscisic acid (ABA) promotes the translocation of COP1 protein to the cytoplasm, subsequently activating the HY5-ABI5 signaling pathway, thereby upregulating ROS accumulation and suppressing seed germination [18]. Multiple studies have further elucidated the impact of ROS imbalance on seed aging. In immature *Helianthus annuus* seeds, attenuated catalase (CAT) activity leads to insufficient ROS scavenging capacity, which deranges the glutathione/oxidized glutathione (GSH/GSSG) redox balance and exacerbates membrane lipid damage, ultimately precipitating seed aging [19]. Similarly, during the aging process of *Triticum aestivum* seeds, the attenuation of antioxidant enzyme activity leads to extensive ROS accumulation, increased protein carbonylation levels, and a diminution in the number of protein bodies within embryonic cells, thereby causing a concomitant reduction in seed viability [20]. Relevant scholars have found that the cytochrome P450 enzyme encoded by the CYP77A4 gene in *Arabidopsis thaliana* can achieve homeostasis between lipid β-oxidation energy supply and ROS production by catalyzing the epoxidation of unsaturated fatty acids. In addition, hydrogen peroxide (H₂O₂) can also achieve dual regulation of ROS homeostasis through transcriptional upregulation of CYP77A4 and cysteine-456 sulfonylation modification of its protein [21]. As signaling molecules, ROS can regulate the transition of seeds between germination and dormancy states. However, when ROS accumulate excessively within seeds, they transform into cytotoxic agents, causing genomic instability such as DNA strand breaks, base modifications, and abnormal chromosome structures, thereby interfering with the stability of genetic information [22–24].

Currently, a comprehensive understanding of the aging mechanism in *Saposhnikovia divaricata* seeds is lacking. Therefore, this study aims to elucidate the aging mechanisms of *Saposhnikovia divaricata* seeds by investigating germination patterns and structural alterations across seeds with varying storage durations. This will be achieved through an integrated approach combining the analysis of ROS molecular dynamics and electrical conductivity values, complemented by transcriptomic and proteomic techniques. Based on these findings, targeted suggestions and countermeasures conducive to seed storage and senescence retardation will be proposed, providing a robust theoretical foundation for the subsequent cultivation and breeding of *Saposhnikovia divaricata* seeds.

## Results

### Dynamics of reactive oxygen species and oxidative damage during seed storage

**Fig. 1:**
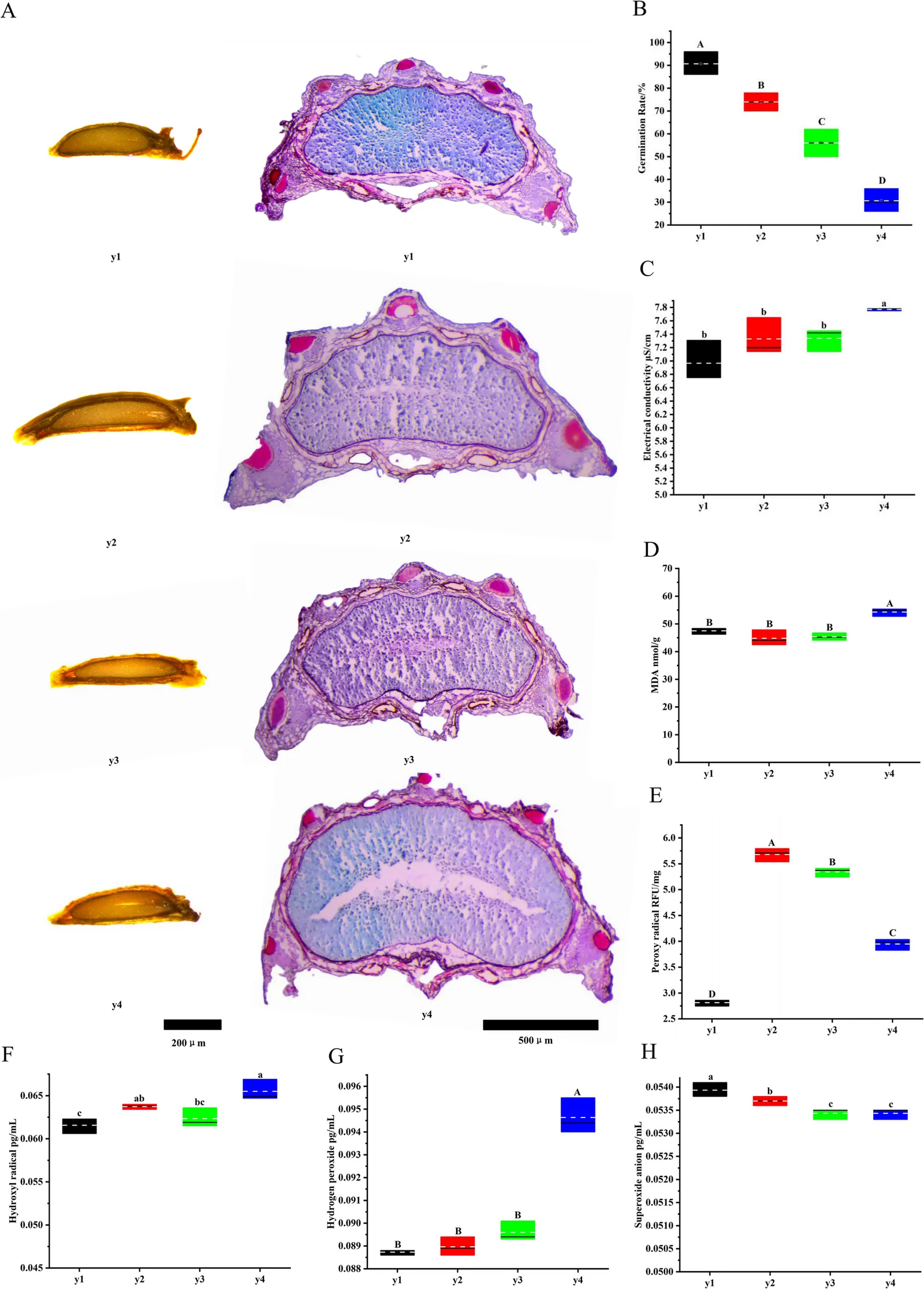
Types of ROS accumulated and damage in *Saposhnikovia divaricata* seeds during storage. **A** Microstructure of *Saposhnikovia divaricata* seeds, (**B**) Germination rate, (**C**) Electrical conductivity, (**D**) Malondialdehyde content, (**E**) Peroxyl radical content, (**F**) Hydroxyl radical content, (**G**) Hydrogen peroxide content, (**H**) Superoxide anion content. (Note: Y1 = 1-year storage, Y2 = 2-year storage, Y3 = 3-year storage, Y4 = 4-year storage. Different lowercase letters indicate significant differences (*P* < 0.05), uppercase letters indicate highly significant differences (*P* < 0.01), and the same letters indicate no significant difference

Microscopic analysis of the seed structure of *Saposhnikovia divaricata* stored over different durations revealed that longitudinal sections of seeds stored for 1 year (Y1) displayed a relatively full embryo with distinct boundaries. As storage time increased, the embryo exhibited a progressive trend of shrinkage and flattening, accompanied by a continuous reduction in morphological integrity. After 4 years of storage (Y4), prominent cavitation was observable within the embryo. Cross-sectional observations indicated that seeds stored for 1 year (Y1) and 2 years (Y2) maintained a relatively intact tissue organization. Although a small number of red-stained structures were present at the embryo edges, no significant cracks or extensive tissue damage were identified, indicating overall structural stability. At 3 years (Y3), embryonic cells displayed marked longitudinal cracks, and localized tissue damage was evident. In seeds stored for 4 years (Y4), structural deterioration was more pronounced, with extensive cavitation observed in both longitudinal and cross-sectional samples (Fig. 1A). With increasing storage duration of *Saposhnikovia divaricata* seeds, the germination rate exhibited a declining trend, with highly significant differences observed among seeds stored for different years. Specifically, the germination rate of seeds stored for 1 year was the highest at 90.67%, while that of seeds stored for 4 years was the lowest at 30.67% (Fig. 1B).

Analysis of the electrical conductivity of *Saposhnikovia divaricata* seeds revealed an increasing trend with prolonged storage duration. The electrical conductivity of seeds stored for 4 years was the highest at 7.77 µS/cm, while that of seeds stored for 1 year was the lowest at 6.97 µS/cm. A significant difference in electrical conductivity was observed between seeds stored for 4 years and those stored for other periods (Fig. 1C). Malondialdehyde (MDA) is a product of lipid peroxidation, and its content directly reflects the extent of cell membrane damage [25]. The MDA content in *Saposhnikovia divaricata* seeds exhibited a biphasic trend, first decreasing and then increasing with extended storage time. The highest MDA level was observed in seeds stored for 4 years (54.3006 nmol/g), while the lowest was in seeds stored for 2 years (44.8025 nmol/g). Significant differences in MDA content were found between seeds stored for 4 years and those stored for other durations (Fig. 1D). Peroxyl radicals are key intermediates in lipid peroxidation and protein carbonylation, and their content reflects the degree of oxidative stress during the early stage of seed aging [26]. The peroxyl radical content in *Saposhnikovia divaricata* seeds exhibited a biphasic trend, initially increasing and subsequently decreasing with storage duration. The lowest level was observed in seeds stored for 1 year (2.8147 RFU/mg), while the highest was in seeds stored for 2 years (5.6800 RFU/mg). Significant differences in peroxyl radical levels were detected among seeds stored for different periods (Fig. 1E). Hydroxyl radicals play a key role in promoting plant cell death, not only causing cell wall relaxation but also attacking proteins, resulting in irreversible biomacromolecular damage [27]. The hydroxyl radical content in *Saposhnikovia divaricata* seeds exhibited a “rise-fall-rise” pattern during storage. The highest hydroxyl radical level was observed in seeds stored for 4 years (0.0655 pg/mL), while the lowest was in seeds stored for 1 year (0.0615 pg/mL), with a significant difference between these two points (Fig. 1F). Hydrogen peroxide (H₂O₂) is also a crucial precursor for the generation of hydroxyl radicals (·OH). It acts as both a potentially toxic substance that needs to be scavenged and a messenger that transmits signals [27]. During the aging process of *Saposhnikovia divaricata* seeds, the H₂O₂ content exhibited an overall increasing trend with storage duration. The highest H₂O₂ concentration was observed in seeds stored for 4 years (0.0946 pg/mL), while the lowest was in seeds stored for 1 year (0.0887 pg/mL), representing a 6.23% decrease. Significant differences in H₂O₂ content were detected between seeds stored for 4 years and those stored for other periods (Fig. 1G). Superoxide anion can be utilized by plants to kill invading viruses, but viruses can also exploit the oxidative environment for their replication [28]. The superoxide anion content in *Saposhnikovia divaricata* seeds exhibited a decreasing trend with storage duration. The lowest levels were observed in seeds stored for 3 and 4 years (0.0534 pg/mL), while the highest was in seeds stored for 1 year (0.0539 pg/mL). The differences among these periods were approximately 0.9% and were statistically significant (Fig. 1H).

### Transcriptomic analysis of *Saposhnikovia divaricata* seeds during storage

**Fig. 2:**
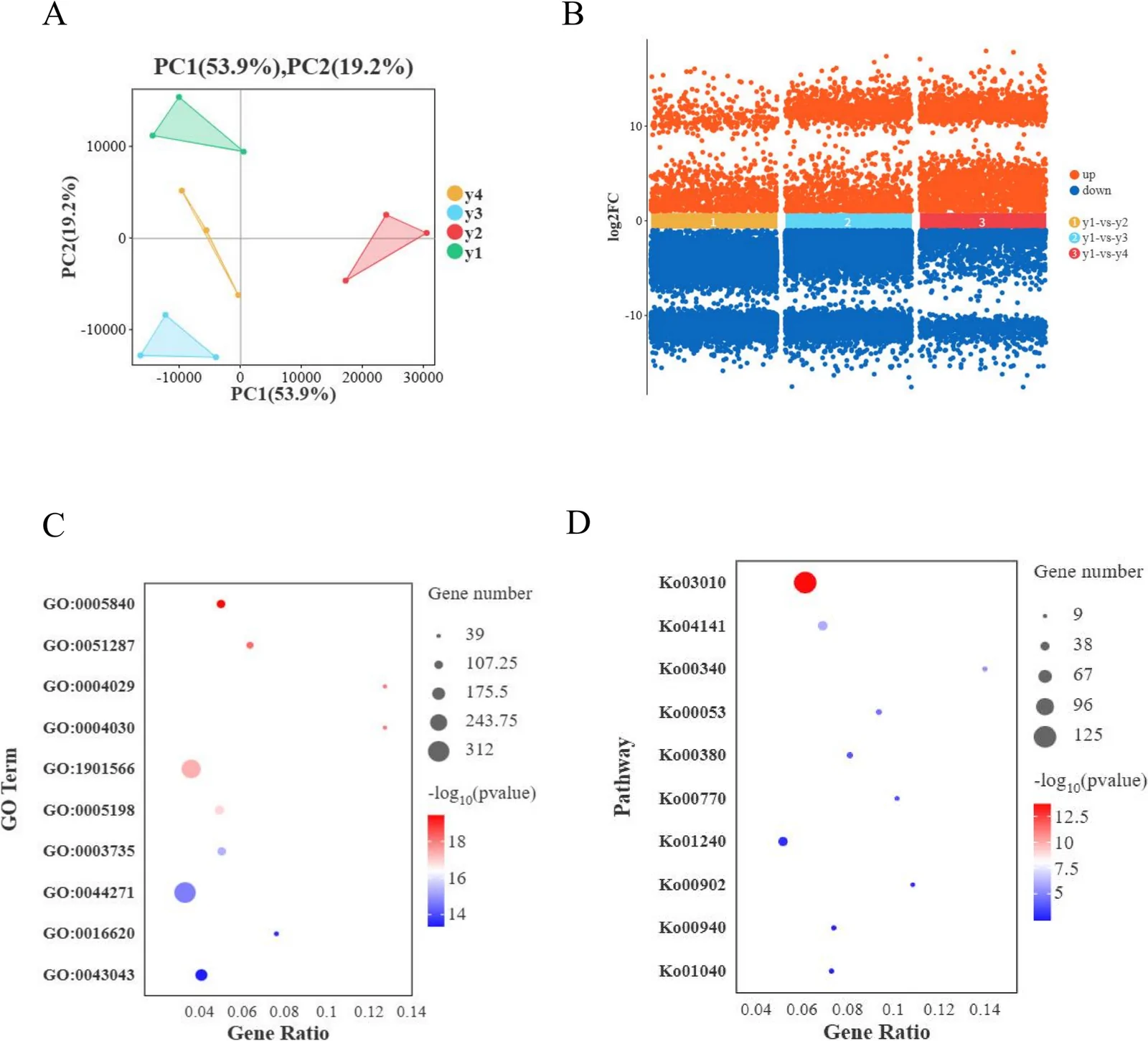
Classification information of the transcriptome of *Saposhnikovia divaricata* seeds. **A** Principal Component Analysis (PCA) of the transcriptome, (**B**) Multi-group differential scatter plot of the transcriptome, (**C**) GO significance bubble plot and STEM original plot of target genes, (**D**) KEGG significance bubble plot of target genes

To investigate the gene expression differences in *Saposhnikovia divaricata* seeds during storage, we analyzed transcriptomic data from seeds stored for 1 to 4 years. A total of 53,784 genes were identified, and the overall distribution of gene expression levels was consistent across all biological replicates ( Appendix A for details). Principal Component Analysis (PCA) was performed on the transcriptomic datasets. The results indicated that Principal Component 1 (PC1) explained 53.9% of the variance, with clear separation among the y1, y2, y3, and y4 groups—reflecting significant transcriptomic differences at various storage stages. Principal Component 2 (PC2) accounted for 19.2% of the variance, and together, PC1 and PC2 explained over 50% of the total variance, confirming the robustness of the analysis (Fig. 2A). Statistical analysis revealed that, compared to the y1 group, the y2, y3, and y4 groups exhibited 1,161, 1,825, and 2,562 upregulated genes, respectively, and 11,470, 10,943, and 2,270 downregulated genes, respectively. In terms of gene counts, the number of downregulated genes was consistently higher than that of upregulated genes. Additionally, with increasing storage duration, the number of upregulated genes showed a slight upward trend (Fig. 2B).

The 1,328 differentially expressed genes (DEGs) utilized for GO and KEGG enrichment analyses in the present study constituted the core gene set with significant differential expression shared across three pairwise comparisons (Y1-vs-Y2, Y1-vs-Y3, Y1-vs-Y4), with the filtering criteria of FDR < 0.05 and |log₂FC| > 2. This core gene set corresponds to molecules that display consistent differential expression throughout the entire aging process of *Saposhnikovia divaricata* seeds during 1 to 4 years of storage (Appendix D). Gene Ontology (GO) molecular function analysis of these 1,328 genes indicated that 107 are involved in the structural components of ribosomes; these genes directly participate in the assembly of ribosomal subunits and, in conjunction with another gene, contribute to intracellular protein synthesis within the ribosome. Additionally, these 107 genes collaborate with a further 15 genes to fulfill structural molecule activity functions—ensuring the maintenance of ribosomal conformational integrity, which enables 162 genes to operate efficiently during peptide biosynthesis. Eighteen genes were enriched in the “organic nitrogen compound biosynthetic process,” 52 genes in the “cellular nitrogen compound biosynthetic process,” and 260 genes were involved in both processes. These pathways are fundamental for cells to synthesize nitrogen-containing small molecules, such as amino acids and nucleotides, which serve as precursors for protein synthesis (peptide biosynthesis) within the ribosomal machinery. An additional 39 genes were associated with “aldehyde dehydrogenase (NAD+/NAD(P)+) activity. " These genes, in conjunction with 11 other genes, participate in oxidoreductase activity, and collectively, the 39 genes also collaborate with 47 additional genes involved in NAD binding(Fig. 2C).

KEGG enrichment analysis of the 1,328 shared DEGs revealed that the first-level categories comprised “Metabolism” (encompassing 8 pathways) and “Genetic Information Processing” (encompassing 2 pathways).

In the “Translation” category, 125 genes were enriched in the ribosome pathway, with 6 genes upregulated and 85 genes downregulated. In the “Folding, Sorting, and Degradation” category, 44 genes were enriched in the “Protein processing in the endoplasmic reticulum” pathway, of which 7 genes were upregulated and 20 genes downregulated. Secretory and membrane proteins synthesized by ribosomes must enter the endoplasmic reticulum for modifications such as folding and glycosylation to produce mature functional proteins. In the “Amino Acid Metabolism” category, 14 genes were enriched in the histidine metabolism pathway and 20 genes in the tryptophan metabolism pathway—both pathways are essential for cellular amino acid biosynthesis. In the “Carbohydrate Metabolism” category, 19 genes were enriched in the ascorbate and aldarate metabolism pathway, which protects cellular structures such as ribosomes and the endoplasmic reticulum from oxidative damage by providing reducing power via ascorbate peroxidase [29]. Among other metabolism-related pathways, 13 genes were enriched in the pantothenate and CoA biosynthesis pathway, and 14 genes in the unsaturated fatty acid biosynthesis pathway. The former supplies ATP necessary for protein synthesis in ribosomes and protein processing in the endoplasmic reticulum, while synergistically regulating lipid metabolism with the latter [30, 31]. Additionally, 41 genes were enriched in the cofactor biosynthesis pathway, involved in processes such as ribosome function, amino acid metabolism, and antioxidant responses. Furthermore, 15 genes were enriched in the phenylpropanoid biosynthesis pathway and 9 genes in the monoterpenoid biosynthesis pathway. These pathways operate synergistically with ascorbate to form an “antioxidant network,” ensuring the proper functioning of protein synthesis and processing pathways(Fig. 2D).

### Proteomic analysis of *Saposhnikovia divaricata* seeds during storage

**Fig. 3:**
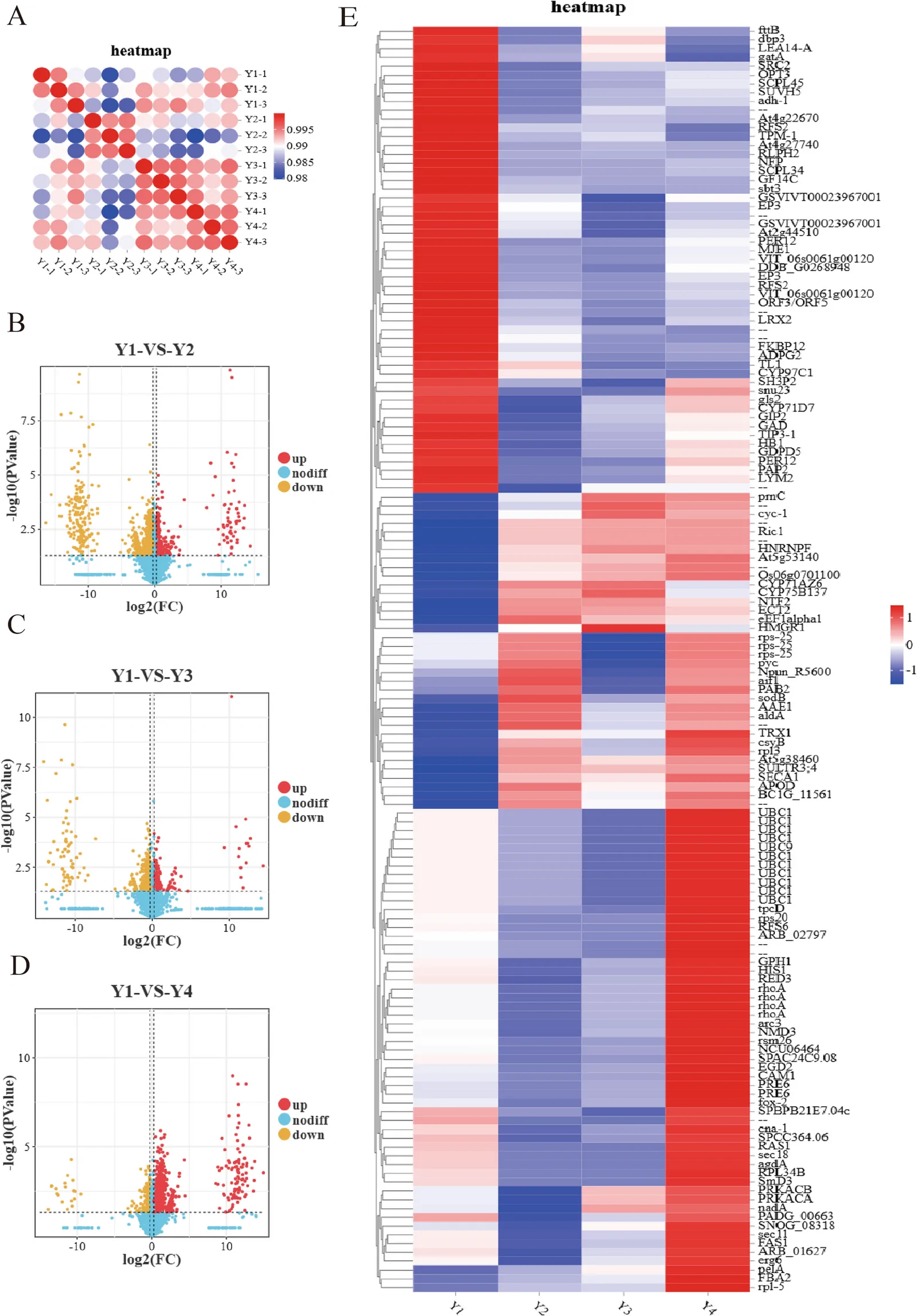
All quantified proteins were used as variables. **A** Sample correlation heatmap. **B** Volcano plot for the Y1 vs. Y2 comparison. **C** Volcano plot for the Y1 vs. Y3 comparison. **D** Volcano plot for the Y1 vs. Y4 comparison. **E** Clustering heatmap of differentially expressed proteins. Criteria: proteins with *P* < 0.05 and fold change (FC) > 1.2 were defined as upregulated proteins; those with FC < 1/1.2 (≈ 0.833) were defined as downregulated proteins

Proteins are translated from RNA, establishing a fundamental correlation between genes and proteins. To investigate changes in protein profiles during the storage of *Saposhnikovia divaricata* seeds, an untargeted proteomic approach was employed. We performed label-free quantitative mass spectrometry to compare the proteomes of seeds stored for 1 to 4 years. A total of 72,767 spectra, 64,423 peptides, and 9,616 proteins were identified (FDR ≤ 0.01; Appendix B for details).The overall distribution of protein intensities was consistent across all biological replicates (Appendix C for details). Data from the correlation heatmap demonstrated that the correlation coefficients among all groups exceeded 0.98, indicating low variability among the samples (Fig. 3A). Further statistical analysis revealed that, compared to the control group Y1, the Y2, Y3, and Y4 groups exhibited 470, 275, and 1,702 upregulated proteins, respectively, and 1,247, 497, and 237 downregulated proteins, respectively. In general, the number of differentially downregulated proteins was lower than that of upregulated proteins, and both categories showed a gradual decline with increasing storage duration (Fig. 3B–E).

**Fig. 4:**
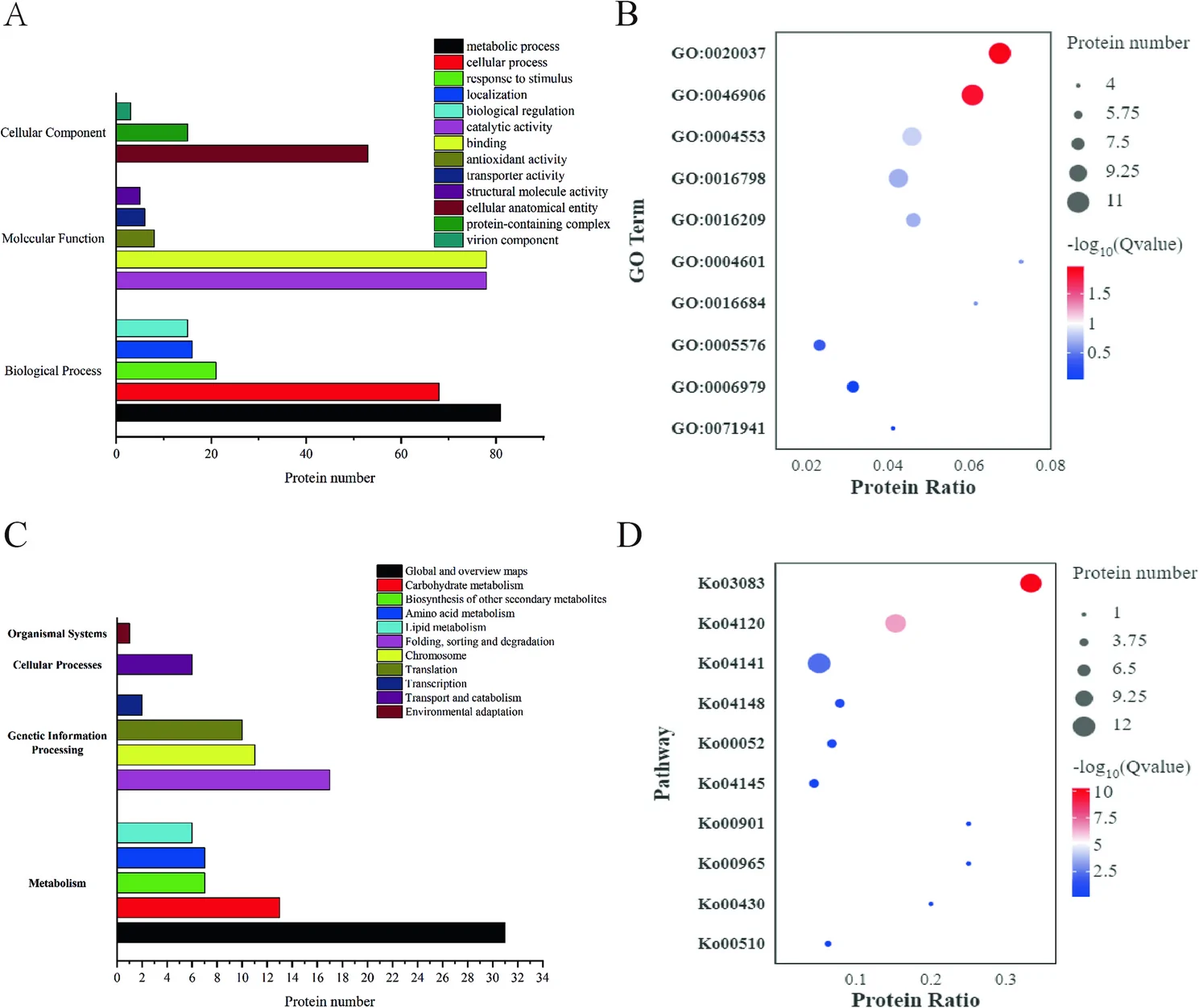
Classification of 144 core differentially expressed proteins (DEPs). **A** GO classification bar plot of 144 core DEPs; (**B**) GO enrichment significance bubble plot of 144 core DEPs; (**C**) KEGG pathway count bar plot of 144 core DEPs; (**D**) KEGG pathway enrichment significance bubble plot of 144 core DEPs. (Note: The 144 core DEPs were defined as the intersection of DEPs identified from three pairwise comparisons (Y2/Y1, Y3/Y1, Y4/Y1) with the filtering criteria of |log₂(Fold Change)| > 1.5 and false discovery rate (FDR) < 0.05. These proteins represent the core functional molecules exhibiting persistently significant abundance alterations throughout the entire seed aging process (1–4 years of storage)

A total of 144 core differentially expressed proteins (DEPs) were screened as the intersection of DEPs identified from the three pairwise comparisons (Y1-vs-Y2, Y1-vs-Y3, Y1-vs-Y4), with the stringent filtering criteria of |log₂(Fold Change, FC)| > 1.5 and false discovery rate (FDR) < 0.05. These DEPs represent the core functional proteins with persistent abundance alterations throughout the entire seed aging process and served as the key subject for subsequent GO/KEGG enrichment and multi-omics integration analyses. This number conforms to the statistical rule of the intersection of multiple group comparisons, whereby stringent criteria lead to a significant reduction in the size of the intersection, thus ensuring the target specificity and reliability of subsequent functional analyses (Appendix E). These proteins were annotated as components of the cytoskeleton, cytoplasm, nucleus, and chloroplasts. GO -based molecular function analysis indicated that “metabolic process” was the most prominent biological process, with 81 proteins enriched in this category, followed by “cellular process,” with 68 proteins. In terms of molecular function, 78 proteins were associated with catalytic activity, and another 78 proteins were involved in binding functions. Additionally, 53 proteins were enriched in “cellular anatomical entity,” primarily representing cell components (Fig. 4A). Analysis of the GO enrichment bubble plot for differentially expressed proteins revealed that 11 proteins were significantly enriched in heme binding and tetrapyrrole binding domains. These proteins play key roles in physiological processes such as respiration and electron transport. Among these, 2 proteins are involved in oxygen binding, exhibiting oxygen-binding capacity in addition to heme function. Furthermore, 10 proteins were collectively enriched in hydrolase activity, specifically acting on O-glycosyl compounds and glycosyl bonds, and are involved in carbohydrate hydrolytic metabolism. An additional 4 proteins were enriched within intrinsic components of the plasma membrane, while 2 proteins were associated with epidermal development processes. Intrinsic membrane components facilitate functions such as cell recognition and signal transduction, supporting the normal development and functional maintenance of epidermal cells. Additionally, 8 proteins were enriched in antioxidant activity, which helps maintain intracellular stability and ensures the proper operation of other cellular pathways (Fig. 4B).

To better understand the metabolic pathways active during the storage of *Saposhnikovia divaricata* seeds, KEGG enrichment analysis was performed on the 144 shared proteins. In the category of metabolic pathways, 31 proteins were distributed across “Global and Overview Maps,” 13 proteins were involved in “Carbohydrate Metabolism,” 7 proteins participated in “Biosynthesis of Other Secondary Metabolites,” another 7 proteins were associated with “Amino Acid Metabolism,” and 6 proteins were involved in “Lipid Metabolism. " In the context of genetic information processing, 17 proteins were enriched in “Folding, Sorting, and Degradation,” 11 proteins in “Chromosomes,” 10 proteins in “Translation,” and 2 proteins in “Transcription. " Regarding cellular processes, 6 proteins were involved in “Transport and Catabolism. " In the organismal systems category, 1 protein was enriched in “Environmental Adaptation” (Fig. 4C). Analysis based on the KEGG significance bubble plot (*p* < 0.05) identified a total of 20 proteins significantly enriched across five pathways. In terms of first-level categories, “Genetic Information Processing” encompassed three pathways, “Cellular Processes” included one pathway, and “Metabolism” involved one pathway. Within the secondary category “Folding, Sorting, and Degradation” under “Genetic Information Processing,” 12 proteins—such as UBC1, UBC9, and GLS2—were collectively enriched in the “Protein Processing in the Endoplasmic Reticulum” pathway. These proteins participate in protein folding and post-translational modifications, ensuring proteome quality. Among them, 11 proteins were also enriched in the “Ubiquitin-Mediated Proteolysis” pathway; these same proteins additionally participated in processes related to the “Chromosomes” category within “Genetic Information Processing” and showed significant enrichment in the “Polycomb Repressive Complex” pathway. The ubiquitin-mediated proteolysis pathway primarily functions in targeted protein degradation and can indirectly regulate gene expression synergistically with the polycomb repressive complex, which inhibits gene transcription through epigenetic modifications [32]. Additionally, four proteins (rhoA) were enriched in the “Efferocytosis” pathway under the secondary category “Transport and Catabolism” within “Cellular Processes. " Abnormal proteins recycled through this pathway are degraded via the ubiquitin-mediated proteolysis pathway. In the “Galactose Metabolism” pathway, under the secondary category “Carbohydrate Metabolism” within “Metabolism,” three proteins (RFS2, RFS6, and agdA) were enriched. The metabolites and energy generated by this pathway support biological processes such as gene regulation and protein processing [33] (Fig. 4D).

### Integrated analysis of transcriptome and proteome

**Fig. 5:**
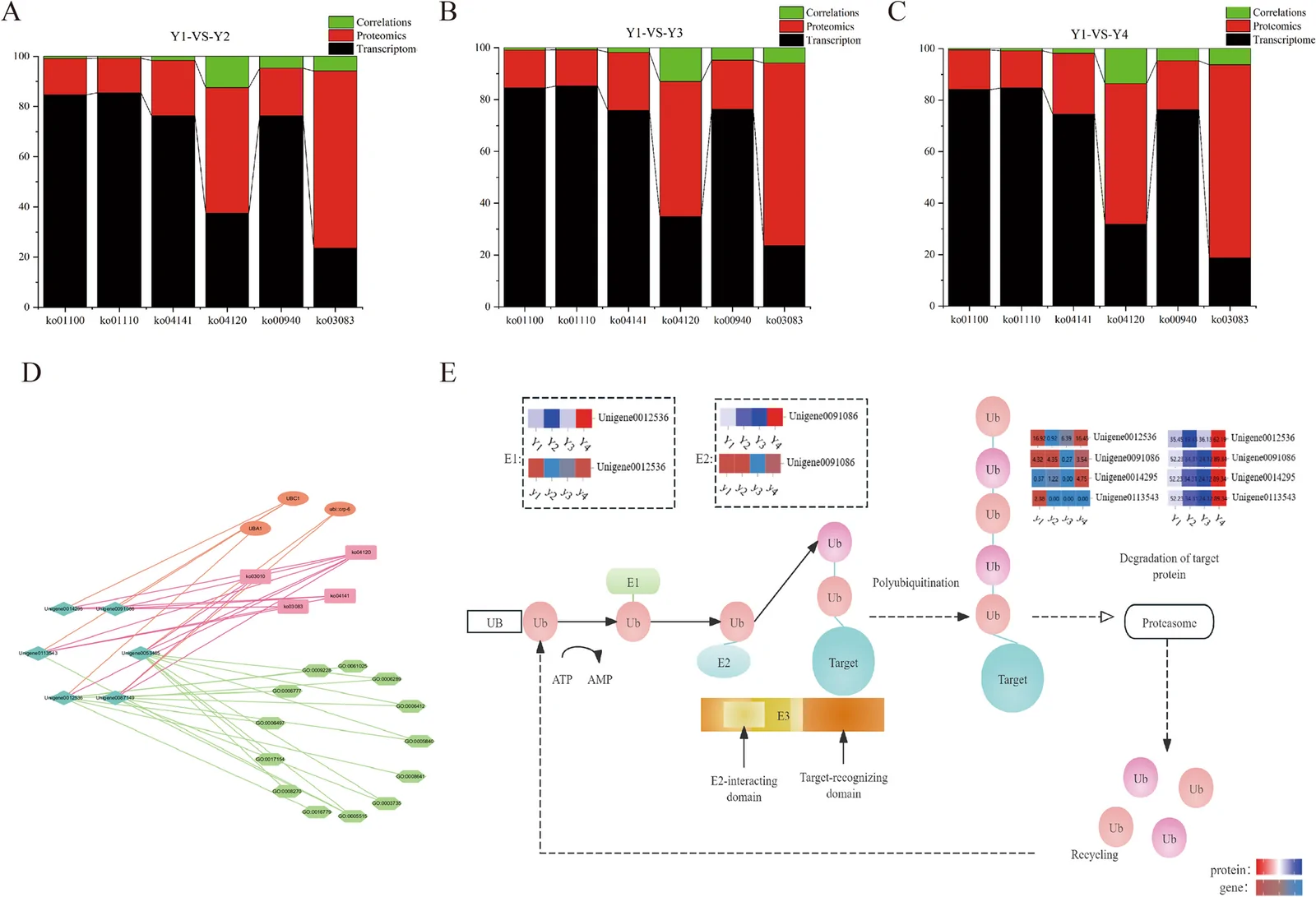
Integrated transcriptome and proteome analysis. **A** Pathway association analysis bar chart for Y1-vs-Y2; (**B**) Pathway association analysis bar chart for Y1-vs-Y3; (**C**) Pathway association analysis bar chart for Y1-vs-Y4; (**D**) Interaction network of differentially expressed genes (DEGs) and differentially expressed proteins (DEPs); (**E**) Schematic diagram of the ubiquitin-mediated proteolysis pathway. (Note: This integrated analysis was based on two core molecular sets: (1) 1,328 core DEGs, which represent the intersection of DEGs from Y2/Y1, Y3/Y1 and Y4/Y1 comparisons with filtering criteria of FDR < 0.05 and |log₂(FC)| > 2; (2) 144 core DEPs, which represent the intersection of DEPs from the same pairwise comparisons with filtering criteria of |log₂(FC)| > 1.5 and FDR < 0.05. Pathway association analysis reveals the synergistic regulation of key biological processes during seed aging by genes and proteins

In this study, an integrated analysis was performed on 1328 core differentially expressed genes (DEGs) and 144 core differentially expressed proteins (DEPs), which were the intersection of DEGs and DEPs identified from the same three pairwise comparisons, respectively. The results showed that the pathway enrichment of both was mainly concentrated in metabolic pathways, biosynthesis of secondary metabolites, protein processing in the endoplasmic reticulum, ubiquitin-mediated proteolysis, phenylpropanoid biosynthesis, and polycomb repressive complex pathways. These 144 core DEPs were subject to persistent regulation throughout the entire seed aging process. Therefore, functional enrichment analysis of these DEPs focused on exploring the persistently activated or inhibited pathways during seed storage, rather than the transient changes in a single storage stage. The ubiquitin-mediated proteolysis pathway could degrade misfolded proteins generated in the protein processing in the endoplasmic reticulum pathway, and specifically participate in the endoplasmic reticulum-associated degradation (ERAD) process (Fig. 5A–C).

To further elucidate the regulatory mechanism of the ubiquitin-mediated proteolysis pathway during the storage of *Saposhnikovia divaricata* seeds, 112 DEGs and 23 DEPs enriched in this pathway were screened out. Subsequently, the Pearson correlation coefficients between these DEGs and DEPs were calculated, with a strong correlation defined as |correlation coefficient| ≥ 0.6 and *p* < 0.05, so as to clarify the expression correlation between genes and proteins during seed storage.

The analysis results indicated that the main biological functions of this pathway were mediated by two genes, namely UBA1 and UBC1. The transcripts and proteins corresponding to Unigene0091086, Unigene0014295 and Unigene0113543 were all associated with the function of the UBC1 gene, while Unigene0012536 was related to the function of the UBA1 gene (Fig. 5D). In addition, the expression levels of transcripts and proteins of these two genes (UBA1 and UBC1) showed a highly significant and strong correlation (Table 1).

**Table 1:** Correlation Analysis Between Genes and Proteins

Gene ID	Protein ID	cor	*p*_value
Unigene0012536	Unigene0014295	0.627	0.029
Unigene0014295	Unigene0014295	0.746	0.005
Unigene0012536	Unigene0091086	0.627	0.029
Unigene0014295	Unigene0091086	0.746	0.005
Unigene0012536	Unigene0113543	0.627	0.029
Unigene0014295	Unigene0113543	0.746	0.005
Unigene0012536	Unigene0012536	0.644	0.024

In the ubiquitin-mediated proteolysis pathway, ubiquitin-activating enzyme E1 (Unigene0012536) first bound to ubiquitin (Ub). This process consumed adenosine triphosphate (ATP) and converted it into adenosine monophosphate (AMP); the released energy promoted the formation of the E1-Ub complex, thus completing the activation of ubiquitin and initiating the ubiquitination cascade reaction. The activated ubiquitin was then transferred to ubiquitin-conjugating enzyme E2 (including Unigene0091086, Unigene0113543 and Unigene0014295), forming the E2-Ub complex responsible for ubiquitin transport. Ubiquitin ligase E3 recognized and bound to the target protein, and simultaneously combined with the E2-Ub complex, covalently attaching ubiquitin to the target protein to form a polyubiquitin chain. The target protein modified by polyubiquitination was recognized and degraded by the proteasome, after which ubiquitin was recycled to participate in the ubiquitination process again.

For genes in the above pathway, Unigene0012536 and Unigene0091086 showed continuous expression in seeds at all storage stages. Transcription of Unigene0113543 was suppressed from the 2nd to 4th storage year, while Unigene0014295 was transcriptionally inhibited in the 3rd year. All four corresponding proteins were consistently expressed in seeds over 1–4 years of storage (Fig. 5E).

### Results of target gene mRNA expression levels in *Saposhnikovia divaricata* seeds during storage

**Fig. 6:**
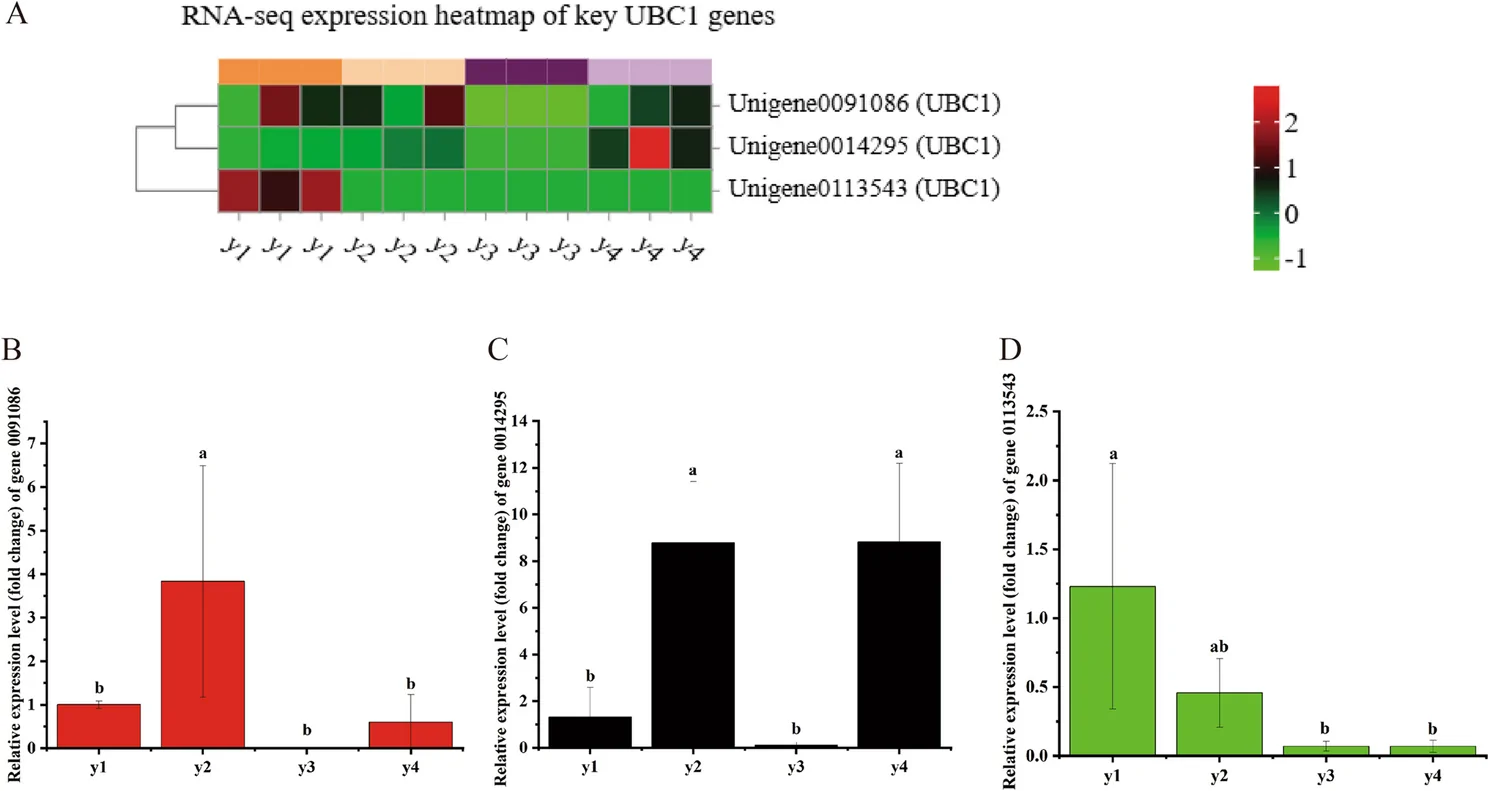
Validation of key UBC1 gene expression using RNA-seq and RT-qPCR. **A** Heatmap showing the log₂-transformed fold changes of three key UBC1 genes across four treatments (y1–y4) from RNA-seq data. **B-D** RT-qPCR validation of the three UBC1 genes.(Note: Expression levels were calculated using the 2^(-ΔΔCt) method, with the y1 group serving as the control. Differences among groups were analyzed using one-way analysis of variance (ANOVA), followed by the Least Significant Difference (LSD) test for post-hoc multiple comparisons. Different lowercase letters indicate significant differences, while the same lowercase letter indicates no significant difference

The results showed that the expression trends of genes in *Saposhnikovia divaricata* seeds from different storage years were consistent with the transcriptome sequencing results, thereby validating the reliability of the transcriptomic data (Fig. 6A–D).

## Discussion

Seeds are susceptible to aging during storage, and germination rate is a key indicator of seed vigor and viability [34]. In this study, the germination rate of *Saposhnikovia divaricata* seeds during storage was monitored, revealing a gradual decline with increasing storage duration, reaching 30.67% in the fourth year. These findings are consistent with observations in Fagus sylvatica seeds, where prolonged storage results in increased cell membrane damage, leading to seed aging and reduced germination capacity [35]. Furthermore, anatomical analyses of *Saposhnikovia divaricata* seeds stored for various periods indicated that seeds stored for 1 year maintained intact and plump embryonic structures, whereas seeds stored for 4 years exhibited structural damage and cavitation. This deterioration is attributable to accumulated cellular damage and energy depletion during storage, which reflect the progressive morphological changes associated with seed aging [36].

Literature indicates that the accumulation of ROS damages cell membrane integrity, decreases seed viability, and consequently inhibits seed germination [37–39]. Electrical conductivity, which reflects the electrolyte leakage from seeds, serves as an indirect indicator of cell membrane damage [40]. In this study, we quantified multiple ROS species (superoxide anion, H₂O₂, hydroxyl radical, peroxyl radical) and MDA to comprehensively characterize the oxidative damage process during seed aging: peroxyl radicals reflect early oxidative stress, hydroxyl radicals indicate irreversible biomolecular damage, H₂O₂ mediates signaling and secondary ROS generation, and MDA quantifies membrane lipid peroxidation. The coordinated changes of these indicators confirm that ROS-induced oxidative damage is a core driver of *Saposhnikovia divaricata* seed aging.

The changes observed in the electrical conductivity of *Saposhnikovia divaricata* seeds during storage revealed a gradual increase with prolonged storage duration, with the value in the 4th year being significantly higher than in earlier years. These results are consistent with findings by Zhang Juan et al., who reported that electrical conductivity in *Eucommia ulmoides* seeds also increased progressively with extending storage time, indicating increased membrane deterioration during seed aging [41].

MDA and peroxyl radicals are byproducts of cellular lipid peroxidation. Both can form covalent cross-links with proteins, leading to damage of protein molecules; lipid peroxidation further deteriorates cell structure and contributes to seed death [42]. In this study, by analyzing the changes in various ROS and related indicators in *Saposhnikovia divaricata* seeds, it was observed that MDA content exhibited an increasing trend with extended storage duration. Notably, the MDA level in seeds stored for the 4th year was significantly higher than in seeds stored for other years. The relatively low MDA level in 2-year stored seeds might be due to the temporary activation of the antioxidant defense system in the early aging stage, while the sharp increase in 4-year stored seeds indicated severe membrane lipid peroxidation caused by excessive ROS accumulation. These findings are consistent with the research by Tian Jianhong et al., who reported a significant increase in MDA content in artificially aged rice seeds correlating with aging progression [43].

Peroxyl radicals showed significant accumulation in *Saposhnikovia divaricata* seeds during the 2nd year of storage. Although their levels in seeds stored for 3 and 4 years were lower than in the 2nd year, they remained extremely significantly higher than in seeds stored for 1 year. The peak peroxyl radical content in 2-year-stored seeds may be responsible for activating the seed antioxidant defense system. This not only resulted in the low malondialdehyde (MDA) content in *Saposhnikovia divaricata* seeds after 2 years of storage, but also contributed to the decrease in peroxyl radical content during the late storage period (3–4 years of storage). Peroxyl radicals can increase protein carbonyl content and, along with other ROS such as hydroxyl radicals, induce oxidative damage to proteins [44]. Analysis of hydroxyl radicals in *Saposhnikovia divaricata* seeds stored over different years revealed that their content increased with extended storage duration, with the highest level observed in seeds stored for 4 years, reaching 0.0655 pg/mL. As the most potent oxidant, hydroxyl radicals are generated in biological tissues through reactions between H₂O₂ and reductive transition metals [45]. Furthermore, the H₂O₂ content in *Saposhnikovia divaricata* seeds increased with storage time, with seeds stored for 4 years exhibiting an extremely significant higher level than those stored for other periods, reaching 0.0946 pg/mL.In the study by Quan V. Vo et al., it was demonstrated that anthocyanins can scavenge ROS and that H₂O₂ can be generated via the protonation of superoxide anions [46]. In *Saposhnikovia divaricata* seeds stored over different years, the superoxide anion content exhibited a gradual decline with extended storage duration. This reduction may contribute to compensatory increases in other ROS, thereby reflecting complex oxidative dynamics during seed aging.

ROS can directly attack biomolecules such as nucleic acids, proteins, and lipids, leading to cellular damage and structural disintegration [47–49]. Transcriptome sequencing of *Saposhnikovia divaricata* seeds stored for varying durations, followed by KEGG enrichment analysis and GO functional annotation of 1,328 genes, revealed that 125 genes were predominantly enriched in the ribosome pathway. Of these, 107 genes were involved in the structural composition of ribosomes. Ribosomes are essential for maintaining fundamental cellular functions and protein homeostasis and play a critical role in regulating cellular stress responses and homeostatic equilibrium [50]. Oxidative stress induced by H₂O₂ can lead to the preferential oxidation of cysteine residues in ribosomal proteins, resulting in damage and functional impairment of the ribosomal pathway [51]. Three antioxidant pathways were identified in *Saposhnikovia divaricata* seeds across different storage periods: ascorbate and aldarate metabolism (19 genes), phenylpropanoid biosynthesis (15 genes), and monoterpenoid biosynthesis (9 genes). Additionally, 44 genes were enriched in the protein processing pathway in the endoplasmic reticulum, which is responsible for folding and modifying proteins synthesized by ribosomes as well as degrading misfolded proteins.

Transcription factors are translated into functional proteins via codon usage, enabling them to perform specific biological roles [52]. Proteomic analysis of *Saposhnikovia divaricata* seeds stored for varying durations revealed that, relative to the control group Y1, the number of upregulated proteins was 470, 275, and 1,702 in Y2, Y3, and Y4, respectively. Conversely, the number of downregulated proteins was 1,247, 497, and 237 in Y2, Y3, and Y4. The number of downregulated proteins decreased progressively with increasing storage duration, while the number of upregulated proteins in Y4 showed a significant increase. GO functional enrichment analysis of the 144 core DEPs (consistently differentially expressed across Y2/Y1, Y3/Y1, Y4/Y1) indicated their involvement in biological processes such as metabolic processes and cellular processes, reflecting persistent functional changes during seed aging. Additionally, these proteins are associated with molecular functions including catalytic activity and binding functions, and they play roles in vital physiological processes such as respiration, electron transport, carbohydrate hydrolysis, antioxidant activity, and plasma membrane signal transduction. KEGG enrichment analysis identified 20 proteins that were significantly enriched across five pathways, including protein processing in the endoplasmic reticulum, ubiquitin-mediated proteolysis, the polycomb repressive complex pathway, efferocytosis, and galactose metabolism. The protein processing in the endoplasmic reticulum pathway is crucial for facilitating correct protein folding, ensuring quality control, directing the degradation of misfolded proteins, and supporting various physiological functions in plants [53]. This pathway involves ERAD, which detects and ubiquitinates misfolded proteins, subsequently transporting them to the cytoplasmic proteasome for degradation. The ubiquitin-mediated proteolysis pathway serves as a key component of ERAD-mediated protein turnover [54]. Notably, 11 proteins were simultaneously enriched in both the protein processing in the endoplasmic reticulum and ubiquitin-mediated proteolysis pathways, indicating their roles in protein degradation processes.

The results of the integrated analysis indicated that *Saposhnikovia divaricata* seeds stored for various durations were primarily enriched in three pathways: protein processing in the endoplasmic reticulum, ubiquitin-mediated proteolysis, and the PRC. Examination of these pathways revealed that the UBC1 gene family was consistently enriched across all three pathways, with the ubiquitin-mediated proteolysis pathway being the primary route through which this gene exerts its functions. As a key E2 ubiquitin-conjugating enzyme linking E1 enzymes to downstream target proteins, the complex expression pattern of UBC1 reflects the intricate regulation of the ubiquitination system [55]. In this study, the expression levels of transcription factors within the UBC1 gene family exhibited variability. Notably, Unigene0091086 was consistently expressed across all storage periods examined in *Saposhnikovia divaricata* seeds. In contrast, Unigene00113543 was expressed in the first year of storage, while its expression was suppressed from the second to the fourth year. In addition, the expression of Unigene0014295 was suppressed in the third year of storage, while it remained normal in all other years. As an E2 ubiquitin-conjugating enzyme, the UBC1 gene family is directly involved in regulating cell apoptosis within the PRC pathway. In Drosophila muscle tissue, knockdown of Effete (eff)—the homolog of UBE2D—resulted in a significant reduction in H2AK119ub1 modifications [56, 57], leading to marked upregulation of pro-apoptotic gene BIM and senescence-associated gene p16INK4a, thereby promoting apoptosis and senescence [58, 59]. In the Pot1bΔ/Δ mouse model with telomere dysfunction, reduced UBE2D expression further exacerbated telomere damage, which induced increased expression of p16INK4a and p21, ultimately accelerating hematopoietic stem cell senescence [60]. The proteins encoded by the transcription factors of the UBC1 gene family were continuously expressed during the four storage periods of *Saposhnikovia divaricata* seeds, exhibiting a dynamic trend characterized by initial downregulation followed by subsequent upregulation. Specifically, in the third year of storage, the expression levels of both the transcripts (mRNA) and corresponding proteins of the UBC1 gene family reached their lowest values throughout the storage cycle. In the fourth year, although there was a partial recovery in the expression levels of both transcripts and proteins, the embryos of *Saposhnikovia divaricata* seeds had already developed a cavitated structure by this time. This structural defect hindered the seeds’ ability to obtain the necessary support for initiating and completing the germination process.

Similarly, within the ubiquitin-mediated proteolysis pathway—a critical pathway—analysis revealed that UBA1 is the key gene/protein that activates UBC1. As the initiator of the ubiquitination cascade, the expression pattern of UBA1 directly influences pathway activity [61]. In this study, the transcripts and proteins of UBA1 were expressed at all stages of storage, with both showing a trend of initial downregulation followed by upregulation, reflecting consistent changes between the two. The expression level of UBA1 began to increase in the second year of storage. This upregulation enhances UBA1’s ubiquitin-activating ability, accelerates the transfer of ubiquitin to E2 enzymes, and improves the efficiency of labeling and degrading damaged proteins—representing an adaptive response of cells to aging stress [62, 63]. In the context of increased damaged proteins resulting from oxidative stress, the precise activation of E2 by UBA1 can mitigate non-specific reactions, efficiently target and degrade abnormal proteins, and maintain the stability of the intracellular environment [64].

Notably, this classic targeted protein degradation mechanism has inherent functional limitations—specifically, it primarily degrades soluble and membrane proteins but cannot efficiently process aggregated proteins or functionally abnormal organelles [65]. This limitation is also reflected in the *Saposhnikovia divaricata* seed system: during the first year of storage, UBA1 and UBC1 transcripts and proteins are stably expressed, establishing an “E1 activation-E2 transfer” pathway [66] to pre-reserve functional molecules for coping with subsequent oxidative stress (ROS accumulation from year 2 to 4). However, transcriptional expression of two core members of the UBC1 gene family, Unigene0113543 and Unigene0014295, was inhibited during storage years 2–4, which led to dysfunction of the ubiquitin-mediated proteolysis pathway in aged seeds. Abnormal assembly of core pathway components, dysregulated enzymatic activity, or impaired autophagic clearance leads to the accumulation of misfolded/damaged proteins (consistent with the upregulation of unfolded protein response-related DEPs in our proteomic data), disrupts intracellular protein homeostasis, and ultimately accelerates seed aging [67, 68]. This mechanism aligns with our phenotypic observations: 4-year stored seeds exhibit severe embryo cavitation, reduced germination rate (30.67%), and increased membrane damage (elevated electrical conductivity and MDA content), highlighting the critical role of UBC1-mediated ubiquitin proteolysis in maintaining seed viability during long-term storage. Based on transcriptomic, proteomic, and physiological data, we constructed a schematic diagram illustrating the seed aging process in *Saposhnikovia divaricata* mediated by ROS-induced stress and UBC1 dysregulation (Appendix F).

This study identified distinct expression patterns of three UBC1 gene family members (Unigene0091086, Unigene0113543, Unigene0014295) in aging *Saposhnikovia divaricata* seeds. However, whether these members exhibit functional redundancy or specific division of labor in regulating the ubiquitin-mediated proteolysis pathway remains unclear. In future research, genetic manipulations such as RNA interference (RNAi) and overexpression can be employed to regulate the expression of each member individually or in combination. This will clarify their specific roles in the degradation of misfolded proteins and the regulation of the polycomb repressive complex pathway during seed aging, thereby further elucidating the molecular mechanism by which the UBC1 gene family modulates seed viability.

## Conclusions

This study systematically elucidated the core mechanism underlying the aging of *Saposhnikovia divaricata* seeds. Accumulation of reactive oxygen species (ROS) including hydroxyl radicals, hydrogen peroxide and peroxyl radicals attacks biomacromolecules such as nucleic acids, proteins and lipids, leading to cellular structural damage and increased electrical conductivity. Pathway enrichment analysis of the 1328 identified differentially expressed genes (DEGs) and 144 differentially expressed proteins (DEPs) revealed that the ubiquitin-mediated proteolysis pathway is the key pathway responsible for the aging and deterioration of *Saposhnikovia divaricata* seeds.Dysregulated transcription of Unigene0113543 and Unigene0014295 may disrupt the interaction between E3 ubiquitin ligase and the E2-Ub complex, affecting polyubiquitination. This change is correlated with impaired clearance of damaged proteins and reduced ubiquitin recycling, suggesting a potential association with seed aging.

As a key regulatory factor in seed aging, the UBC1 gene family provides a novel perspective for deciphering the aging mechanisms of medicinal plant seeds. This study not only clarifies the intrinsic regulatory pathway of *Saposhnikovia divaricata* seed aging, but also provides a theoretical basis for optimizing seed storage strategies and developing seed viability maintenance technologies, which holds great significance for promoting the sustainable development of the medicinal plant cultivation industry.

## Materials and methods

### Plant materials

The seeds of *Saposhnikovia divaricata* (Turcz.) Schischk. used in this study were collected in 2020 from the pastoral area of Ewenki Banner, Hulunbuir City, Inner Mongolia Autonomous Region (119.75° E, 49.14° N). Seed samples were gathered from harvested forage piles with the permission of local herdsmen. All materials were morphologically identified as authentic *Saposhnikovia divaricata* seeds by Professor Shuangli Liu from Jilin Agricultural University. Physical quality tests revealed that the contents of sediment, damaged grains and weed seeds met the experimental requirements. The initial seed purity was above 90%, and the initial moisture content was 9.622%.

All seeds were sealed in non-porous polyethylene (PE) bags and stored under constant dark conditions at 20 ± 5 ℃ with a relative humidity of 30%–40%. Uniform storage conditions were maintained throughout the experiment to eliminate errors caused by environmental heterogeneity. Starting from 2021, seeds were randomly sampled once a year. Each treatment included three biological replicates with 50 g seeds per replicate, corresponding to a total of 150 g seeds per group. Seed samples with storage durations of 1, 2, 3 and 4 years were obtained successively. All sampled seeds were immediately preserved in an ultra-low temperature refrigerator at − 80 ℃ for subsequent analyses.

## Methods

### Quantification of ROS content and seed damage during storage

#### Microstructural analysis of *Saposhnikovia divaricata* seeds

For each storage group, 5 g of seeds were randomly weighed with three biological replicates (15 g of seeds total per storage group). After homogenizing the samples within the same storage group, 3 g of *Saposhnikovia divaricata* seeds were randomly selected for the experiment. Longitudinal sections were prepared using free-hand dissection, while cross-sections were made via paraffin sectioning. Both types of sections were observed under a stereomicroscope (Leica, Germany) [69].

#### Germination rate assessment

Three biological replicates were set for each storage group, with 50 seeds per replicate (150 seeds total per group). The paper germination method was adopted: seeds were evenly placed on moist filter paper in Petri dishes (9 cm diameter), with filter paper kept continuously moist to avoid desiccation. The germination test was conducted in a constant temperature chamber at 20 ± 5 °C under dark conditions (relative humidity 60–70%), consistent with the natural germination environment of *Saposhnikovia divaricata* seeds. Germination was recorded daily for 30 consecutive days, with seeds considered germinated when the radicle protruded through the seed coat and reached a length of ≥ 2 mm [69].

#### Electrical conductivity measurement

Samples (1 g) of seeds from each biological replicate were immersed in 50 mL distilled water (ultrapure grade) in a sterile beaker. After vigorous shaking for 30 s to ensure full contact between seeds and water, the mixture was incubated at 25 °C in a dark incubator for 24 h (without disturbance). Following a second vigorous shake for 30 s, leachate conductivity was measured using a DDS-11 A conductivity meter (Leici, China). Three biological replicates were performed per storage group.

#### Reactive oxygen species (ROS) quantification

Superoxide anion (Shanghai Youxuan Biotechnology Co., Ltd., China), hydrogen peroxide (Shanghai Youxuan Biotechnology Co., Ltd., China), hydroxyl radical (Shanghai Youxuan Biotechnology Co., Ltd., China), peroxyl radical (Shanghai Beibo Biotechnology Co., Ltd., China), and malondialdehyde (Beijing Solarbio Science & Technology Co., Ltd., China) contents were quantified using an automated microplate reader (Thermo Scientific, USA) and a fluorescence spectrophotometer (Hitachi, Japan), following manufacturers’ protocols.

For each storage group, 10 g of seeds were randomly weighed with three biological replicates (30 g of seeds total per storage group). After homogenizing the samples within the same storage group, three subsamples were randomly selected according to the sample dosage required by the kits for the determination of reactive oxygen species (ROS)-related indicators (superoxide anion, hydrogen peroxide, hydroxyl radical, peroxyl radical) and malondialdehyde (MDA) content. Three technical replicates were performed for each subsample to ensure the accuracy of the detection results.

### Determination of transcriptomics during storage of *Saposhnikovia divaricata* seeds

#### RNA extraction and quality control

Total RNA was extracted from 100 mg of frozen *Saposhnikovia divaricata* seed tissue (three biological replicates per storage year) using the OmniPlant RNA Kit (DNase I) (CWBIO, China; Catalog No.: CW2598S) following the manufacturer’s standard protocol with no modifications. Briefly, seed tissue was ground into a fine powder in liquid nitrogen and immediately mixed with 500 µL Buffer RLS (supplemented with 20 µL β-mercaptoethanol per 1 mL Buffer RLS as recommended by the kit instructions) for thorough homogenization. The lysate was centrifuged at 13,400×g (12,000 rpm) at 4 °C for 2 min, and the supernatant was transferred to a Spin Column FS for filtration (13,400×g, 4 °C, 1 min) to remove insoluble debris. An equal volume of 0.5× anhydrous ethanol was added to the filtrate, and the mixture was loaded onto a Spin Column RM for RNA adsorption. After washing with 350 µL Buffer RW1, on-column DNase I digestion was performed by adding 80 µL DNase I reaction mixture (52 µL RNase-Free Water, 8 µL 10×Reaction Buffer, 20 µL DNase I) and incubating at 20–30 °C for 15 min to eliminate genomic DNA contamination. The column was then washed twice with 500 µL Buffer RW2 (pre-supplemented with anhydrous ethanol) and centrifuged at 13,400×g for 2 min to remove residual ethanol. Purified RNA was eluted with 30–50 µL RNase-Free Water (room temperature incubation for 2 min followed by centrifugation at 13,400×g, 4 °C, 1 min) and stored at − 70 °C to prevent degradation. RNA integrity and purity were verified by 1.2% agarose gel electrophoresis (intact 28 S and 18 S rRNA bands) and a Nanodrop 2000 spectrophotometer (Thermo Scientific, USA) with A260/A280 ratios of 1.8–2.0 and A260/A230 ratios ≥ 2.0, ensuring suitability for subsequent library construction.

Agarose gel electrophoresis was used to verify the integrity of the nucleic acid sample, check for degradation, and detect protein contamination. Intact total RNA shows main ribosomal RNA (rRNA) bands after electrophoresis.

#### Library preparation and sequencing

mRNA Capture Beads (Illumina, USA) were equilibrated to room temperature from 4 °C. Total RNA (1 µg) was adjusted to 50 µL with nuclease-free water and incubated with beads (65 °C, 5 min) for poly(A)+ mRNA enrichment. Following two wash cycles, fragmented mRNA was generated using Frag/Prime Buffer (NEBNext, USA) at 94 °C for 8 min. Fragment size distribution was analyzed by Agilent 2100 Bioanalyzer (Agilent Technologies, USA).

First-strand cDNA synthesis was performed in a thermal cycler (Bio-Rad, USA) under: 25 °C (10 min), 42 °C (15 min), and 70 °C (15 min) with lid heating at 105 °C. Second-strand synthesis proceeded at 16 °C (30 min) and 72 °C (15 min). Adapter ligation (T4 DNA Ligase, NEB, USA) was conducted at 20 °C for 15 min.

Adapter-ligated DNA was purified twice with AMPure XP beads (Beckman Coulter, USA) and 80% ethanol. PCR amplification used KAPA HiFi HotStart ReadyMix (Roche, Switzerland) under: 98 °C (1 min); 12 cycles of 98 °C (10 s)/60°C (75 s); final extension at 72 °C (5 min). Libraries were quantified using DNA 1000 Assay Kit (25–1000 bp detection, 0.1–50 ng/µL) or High Sensitivity DNA Assay Kit (50–7000 bp detection, 5–500 pg/µL) (Agilent Technologies, USA) and sequenced on NovaSeq 6000 (Illumina, USA) with 150 bp paired-end reads.

### Proteomic analysis of stored *Saposhnikovia divaricata* seeds

#### Protein extraction and digestion

Seed proteins from four storage durations were processed using the iST Sample Preparation Kit (PreOmics, Germany), with three replicates per storage year. Aliquots (50 µg protein) were lysed in 50 µL lysis buffer (95 °C, 10 min, 1000 rpm thermomixer agitation). After cooling to 25 °C, trypsin digestion solution (1:20 w/w) was added for proteolysis (37 °C, 2 h, 500 rpm). Reactions were quenched with 20 µL stop buffer. Peptides were desalted using iST cartridges and eluted with two aliquots of 100 µL elution buffer. Purified peptides were lyophilized and stored at − 80 °C prior to LC-MS/MS analysis.

#### DirectDIA mass spectrometry analysis

Lyophilized peptides were reconstituted in mobile phase A (0.1% formic acid) and analyzed using a timsTOF Pro 2 mass spectrometer (Bruker Daltonics GmbH, Germany) coupled to an UltiMate 3000 RSLCnano system (Thermo Fisher Scientific, USA). Samples (200 ng) were loaded onto an Aurora Series C18 column (15 cm × 75 μm, 1.7 μm, 120 Å; IonOpticks, Australia) maintained at 50 °C. Separation was achieved with a 60-min gradient (400 nL/min flow rate): 4–28% B (0.1% formic acid in acetonitrile) over 25 min, 28–44% B in 10 min, 44–90% B in 10 min, held at 90% B for 7 min, then re-equilibrated to 4% B for 8 min.

Mass spectrometry operated in data-independent acquisition parallel accumulation-serial fragmentation (diaPASEF) mode with m/z 349–1229 scanning range (40 Da isolation windows). Collision energy ramped from 20 eV to 59 eV across ion mobility values (1/K₀ 0.6–1.6 Vs/cm²) during PASEF-MS/MS scans.

#### Integrated analysis of transcriptome and proteome

To ensure the reliability of Pearson Correlation Coefficient (PCC) calculation in the integrated transcriptome-proteome analysis, the following data preprocessing steps were performed to guarantee data comparability:①. Transcriptomic data preprocessing: Low-expression genes with FPKM < 1 were filtered out to eliminate background noise. The remaining FPKM values were subjected to log₂(FPKM + 1) transformation to normalize data distribution and reduce the impact of extreme values. ②. Proteomic data preprocessing: Low-confidence proteins with FDR > 1% were excluded. Missing values were imputed using the minimum value method to retain valid data dimensions. The quantitative values were transformed by log₂(quantitative value + 1) to align the data distribution with that of the transcriptome. Finally, Local normalization was performed using Pulsar software to eliminate background differences between samples.③. Cross-omics data matching: Genes and proteins were matched based on Unigene IDs derived from transcriptome de novo assembly (Sect. 2.3.2), ensuring a one-to-one correspondence between molecules in the transcriptome and proteome.

#### RT-qPCR detection of target gene mRNA expression levels in *Saposhnikovia divaricata* seeds during storage

Total RNA was extracted from 100 mg of ground *Saposhnikovia divaricata* seed samples with different storage durations using the SPARKeasy Plant RNA Kit (Catalog No.: AC0305, SparkJade). RNA quality was verified by a Nanodrop 2000 spectrophotometer and 1.2% agarose gel electrophoresis, with clear rRNA bands confirming RNA integrity. Qualified total RNA was reverse-transcribed into complementary DNA (cDNA) following the protocol of the SPARKscript Ⅱ All-in-one RT SuperMix for qPCR (With gDNA Eraser) (Catalog No.: AG0305, SparkJade) on an Applied Biosystems ProFlex PCR System (Applied Biosystems, USA).

RT-qPCR was performed using the 2× Universal SYBR Green qPCR Mix (Catalog No.: AH0105, SparkJade) on a Roche LightCycler^®^ 96 real-time fluorescent quantitative PCR instrument (Roche, Switzerland). The reaction conditions were as follows: pre-denaturation at 95 °C for 30 s; 40 cycles of denaturation at 95 °C for 5 s, annealing at 60 °C for 10 s, and extension at 72 °C for 20 s; followed by melting curve analysis (95 °C for 15 s → 60 °C for 1 min → 95 °C for 15 s) to verify amplification specificity. The 10 µL reaction system consisted of 1.0 µL cDNA template, 0.2 µL each of 10 pmol/µL forward and reverse primers, 5.0 µL 2× Universal SYBR Green qPCR Mix, and RNase-Free H₂O to bring the volume to 10 µL.

The relative mRNA expression levels of target genes were calculated using the 2⁻ΔΔCt method, with glyceraldehyde-3-phosphate dehydrogenase (GAPDH) serving as the internal reference gene. Each sample was analyzed in three replicates to ensure reproducibility.

### Bioinformatic analyses

#### Transcriptomic bioinformatic workflow

##### Raw data processing

Raw sequencing reads were quality-filtered using fastp v0.23.4 with parameters: -q 20 -n 10 -l 50 (filtering reads with > 50% bases having Phred quality score ≤ 20, >10% ambiguous N bases, or length < 50 bp), and adapter sequences were automatically removed. RNA integrity was verified by 1.2% agarose gel electrophoresis and a Nanodrop 2000 spectrophotometer (Thermo Scientific, USA), with A260/A280 ratios of 1.8–2.0 and A260/A230 ratios ≥ 2.0.

##### De novo assembly

Clean reads were assembled into non-redundant Unigenes using Trinity v2.15.1 with default parameters (min_kmer_cov = 2). Redundant sequences were eliminated to construct a reference transcriptome for downstream analyses.

##### Expression quantification

Clean reads were mapped back to the assembled Unigenes and quantified using RSEM v1.3.3. Transcript abundance was measured in Transcripts Per Million (TPM) for cross-sample comparison, while raw read counts were also generated for subsequent differential expression analysis.

##### Differential expression analysis

Raw read counts were imported into DESeq2 v1.38.3 (R v4.2.0) for differential expression analysis. The workflow included: ① normalization of raw counts; ② calculation of raw *p*-values based on the negative binomial distribution model; ③ multiple hypothesis testing correction to obtain the false discovery rate (FDR). Differentially expressed genes (DEGs) were defined as those with FDR < 0.05 and |log₂(Fold Change, FC)| > 2.

##### PCA and visualization

Principal Component Analysis (PCA) was performed using the prcomp function in R v4.2.0 based on the TPM matrix of all genes, with scale=TRUE for data standardization. PCA plots were generated using ggplot2 v3.4.2.

##### Volcano plot construction

Volcano plots for pairwise comparisons (Y1-vs-Y2, Y1-vs-Y3, Y1-vs-Y4) were generated using ggplot2 v3.4.2, with the x-axis representing log₂(FC) and the y-axis representing -log₁₀(FDR). DEGs were highlighted in red (upregulated) and blue (downregulated), while non-DEGs were shown in gray.

##### GO and KEGG enrichment analysis

Enrichment analysis was performed using clusterProfiler v4.6.2 (R v4.2.0) against the Gene Ontology (GO) database (http://www.geneontology.org/) and the Kyoto Encyclopedia of Genes and Genomes (KEGG) Pathway database. The hypergeometric test was used to calculate enrichment p-values. For GO terms, the Bonferroni correction was applied, with corrected *p* ≤ 0.05 considered significant. For KEGG pathways, the Benjamini-Hochberg (BH) correction was used, with FDR ≤ 0.05 considered significant. Enrichment bubble plots and bar plots were generated using ggplot2 v3.4.2.

#### Proteomic bioinformatic workflow

##### Data quality control and quantification

Raw DIA-MS data were quality-controlled using QuiC software (Biognosys) to ensure sample reproducibility. Internal Retention Time (iRT) calibration was performed using the Biognosys iRT Kit to standardize retention times. Protein identification and quantification were conducted using Spectronaut 18 software (Biognosys AG) against a protein database derived from the transcriptome-assembled Unigenes. Key parameters were: trypsin as the digestive enzyme; carbamylation of cysteine (Cys) as a fixed modification; oxidation of methionine (Met) as a variable modification. Only peptides with a Qvalue (FDR) ≤ 1% were retained for reliable quantification. Protein abundance was calculated using the MaxLFQ algorithm, and local normalization was performed via Pulsar software to eliminate inter-sample quantitative biases.

##### Differential expression analysis

Protein abundance data were analyzed using R v4.2.0. Student’s t-test was performed to obtain raw p-values, which were then corrected using the BH method to generate FDR values. Differentially expressed proteins (DEPs) were defined as those with FDR < 0.05 and |log₂(FC)| > 1.5.

##### Heatmap construction

Sample correlation heatmaps and DEP clustering heatmaps were generated using pheatmap v1.0.12 (R v4.2.0). Sample correlations were calculated based on Pearson’s correlation coefficient of protein abundance. DEP clustering was performed using Euclidean distance and complete linkage clustering. For multi-sample heatmaps, data were normalized using z-score, and quantitative values were log₁₀-transformed for visualization.

##### Volcano plot construction

Volcano plots for pairwise proteomic comparisons were generated using ggplot2 v3.4.2, following the same coordinate definitions and color schemes as the transcriptomic volcano plots.

##### GO and KEGG enrichment analysis

The same databases (GO and KEGG) and analytical tools (clusterProfiler v4.6.2) as used for the transcriptomic analysis were employed. The hypergeometric test and BH correction were applied, with FDR ≤ 0.05 as the threshold for significantly enriched GO terms and KEGG pathways.

#### Transcriptome-proteome integration analysis

##### Cross-omics matching

Genes and proteins were matched based on Unigene IDs derived from the transcriptome de novo assembly to ensure a one-to-one correspondence between molecules in the two omics datasets.

##### Pearson Correlation Coefficient (PCC) analysis

Prior to PCC calculation, both datasets underwent preprocessing to ensure comparability. For transcriptomic data, genes with FPKM < 1 were filtered out, and the remaining values were transformed using log₂(FPKM + 1). For proteomic data, proteins with FDR > 1% were excluded; missing values were imputed using the minimum value method; the quantitative values were log₂-transformed and normalized via the Local normalization method in Pulsar software. PCC between the preprocessed transcriptomic (log₂(FPKM + 1)) and proteomic (normalized log₂(quantitative value + 1)) data was calculated using the cor function in R v4.2.0. A strong correlation was defined as |PCC| ≥ 0.6 and *p* < 0.05.

##### Pathway association analysis

Four-quadrant plots and nine-quadrant plots were generated using R v4.2.0 to visualize the consistency of expression trends between DEGs and DEPs. Pathway association bar charts were constructed to reflect the synergistic regulation of key biological processes by genes and proteins during seed aging.

### Statistical analysis

All physiological data (germination rate, electrical conductivity, ROS content, MDA content) were compiled using Excel and analyzed with IBM SPSS Statistics 27.0. Data are presented as the mean ± standard deviation (SD) of three independent biological replicates. One-way analysis of variance (ANOVA) was performed to test for overall group differences, followed by the Least Significant Difference (LSD) test for post-hoc multiple comparisons. Differences were considered statistically significant at *P* < 0.05 and highly significant at *P* < 0.01.

#### Omics data statistics

Differential expression analyses for transcriptomics (DESeq2 v1.38.3) and proteomics (Student’s t-test + BH correction) were performed as described in the “Bioinformatic Analyses” section. Correlation analyses (sample correlation and PCC between transcriptome and proteome) were based on Pearson’s correlation coefficient, with significance determined using the cor.test function in R v4.2.0.

#### Visualization statistics

All statistical graphs (PCA plots, volcano plots, heatmaps, enrichment plots, quadrant plots) were generated using R packages (ggplot2 v3.4.2, pheatmap v1.0.12) with embedded statistical results (e.g., P values, FDR, correlation coefficients) to ensure transparency and reproducibility.

## Supplementary Information

Supplementary Material 1.

Supplementary Material 2.

## Data Availability

The raw transcriptome sequencing datasets generated in this study have been deposited in the Genome Sequence Archive (GSA) database (accession:CRA033939), and the corresponding proteomic raw data were submitted to the OMIX database (accession:OMIX017442).

## Abbreviations

ABA: Abscisic acid
AMP: Adenosine Monophosphate
ATP: Adenosine Triphosphate
CAT: Catalase
CoA: Acetyl Coenzyme A
DEGs: Differentially expressed genes
DEPs: Differentially expressed proteins
ERAD: Endoplasmic reticulum-associated degradation
GO: Gene Ontology
GSH/GSSG: Glutathione/oxidized glutathione
H₂O₂: Hydrogen peroxide
KEGG: Kyoto Encyclopedia of Genes and Genomes
MDA: Malondialdehyde
NAD(P)+: Nicotinamide Adenine Dinucleotide Phosphate
NAD+: Nicotinamide Adenine Dinucleotide
PC1: Principal Component 1
PC2: Principal Component 2
PCA: Principal Component Analysis
PCC: Pearson Correlation Coefficient
PRC: Polycomb repressive complex
ROS: Reactive oxygen species
Ub: Ubiquitin
UBA1: E1 ubiquitin-activating enzyme
UBC1: E2 ubiquitin-conjugating enzyme
