# Supplementary material for: Disruption of the ubiquitin-mediated proteolysis pathway: a study of seed aging in Saposhnikovia divaricata caused by UBC1 gene family suppression
Source: BMC Plant Biol. 2026 Jun 23;26:1539. doi: 10.1186/s12870-026-09258-3 (PMC13551917; doi:10.1186/s12870-026-09258-3)
Supplement: Supplementary file 1 [file 12870_2026_9258_MOESM1_ESM.zip › Supplementary Information.docx]

**Supplementary Material 1: Additional file 1:**

**Appendix A:**Violin plots illustrating the distribution of gene expression levels

**Supplementary Material 2: Additional file 2:**

**Appendix B:**Protein identification statistics according to a filtering standard of FDR ≤ 0.01.

**Supplementary Material 3: Additional file 3:**

**Appendix C:**Violin plot illustrating protein expression levels
